# A Spectrum of Selves Reinforced in Multilevel Coherence: A Contextual Behavioural Response to the Challenges of Psychedelic-Assisted Therapy Development

**DOI:** 10.3389/fpsyt.2021.727572

**Published:** 2021-12-07

**Authors:** Henry J. Whitfield

**Affiliations:** ^1^Regent's University London, London, United Kingdom; ^2^Mindfulness Training Ltd, London, United Kingdom; ^3^Department of Neuropsychology and Psychopharmacology, Faculty of Psychology and Neuroscience, Maastricht University, Maastricht, Netherlands

**Keywords:** psilocybin-assisted psychotherapy, acceptance and commitment therapy, psychological flexibility, self-perspective taking, psychedelic, internal family systems, relapse prevention, integration

## Abstract

Psychedelic-assisted therapy research for depression and PTSD has been fast tracked in the United States with the Food and Drugs Administration (FDA) granting breakthrough designations for MDMA (post-traumatic stress disorder) and psilocybin (major depressive disorder). The psychotherapeutic treatments accompanying these psychedelics have not been well-studied and remain controversial. This article reviews the challenges unique to psychedelic-assisted therapy and introduces a newly optimised psychological flexibility model that adapts Contextual Behavioural Science (CBS)/Acceptance and Commitment Therapy (ACT) to those multiple challenges, including ego inflation, traumatic memories, and the perceived presence of *entities*. A methodology aligned with biological mechanisms, psychological processes and therapeutic contexts may be advantageous for improving outcomes. This model expands ACT by integrating practices and data from psychedelic-assisted therapy research into a Contextual Behavioural Science framework, allowing both fields to inform each other. Psychological flexibility processes are questioned and adapted to a psychedelic context, and interventions that operationalise these processes are considered. The principle through-line of the paper is to consider varied constructs of Self, as understood by these fields, and integrates respective elements of varied self-models, interventions and data into a Spectrum of Selves model for psychedelic-assisted therapy. Secondly the paper examines how to select and retain new self-perspectives and their corresponding behaviours systemically, drawing from evolutionary science principles. A case example of such behavioural reinforcement is provided, as well as a psychedelic integration checklist to guide the practical implementation of such an approach. This method can enable a coherent therapeutic framework with clear operational relationships between (1) problematic behaviour patterns that an individual wishes to address (2) the guided psychedelic experiences of that individual, and (3) the barriers to maintaining any changes, thus increasing theoretical-practical coherence, broadening treatment benefits and reducing relapse in psychedelic-assisted therapy. Research questions for further developing a CBS-consistent psychedelic-assisted therapy are offered.

## Introduction

*No one can whistle a symphony. It takes a whole orchestra to play it*.

—H.E. Luccock

Psychedelic-assisted therapy is a psychopharmacological intervention combining a psychedelic compound with a therapeutic context (1). Unlike purely psychological interventions, psychedelic-assisted therapy is usually considered to have three distinct phases of treatment: (1) psychotherapeutic preparation before medication session, (2) a guided psychedelic medication session with psychological support, and (3) a psychotherapeutic integration therapy to help consolidate gains (2). Randomised control trials have demonstrated effect sizes generally greater than those of current treatments, for depressive disorders (3, 4), addictions (5), distressed cancer patients (6, 7), and PTSD (8). These latter researchers concur that the psychotherapeutic component is of particular importance for safety and maximising therapeutic gains. Psychedelics are not recommended as purely pharmacological interventions. However, integrated psychotherapeutic frameworks grounded in empirically supported processes throughout the three phases of psychedelic-assisted therapy, are only just beginning to emerge, and mainly focus on the preparation and integration phases (9–11).

### Consensus and Debate in Psychedelic-Assisted Therapy

There is some consensus regarding the preparation needed before administering a psychedelic for therapeutic purposes. Psychoeducation and experiential exercises are often employed to help the participant trust and enter the process with an open attitude (7, 11, 12). A lack of feeling safe during the psychedelic experience could either block the therapeutic process or even result in a paranoid experience of negative-therapeutic value [(13), p. 95].

Regarding the guided medication and integration (phases 2 and 3), there is more debate. For example when during a medication session should a psychedelic therapist be directive rather than the commonly recommended approach of trusting a person's “Inner healing intelligence” [(14), p. 10]. An ACT-informed psychedelic study protocol also leant towards “supportive” and “non-directive” guide-therapists with emotional direction coming mainly from a musical playlist during the medication phase [(15), p. 10]. The same study favoured an “open, flexible, humanistic approach” over a “structured, directive approach” more generally [(11), p. 99]. Similarly, another ACT-informed psychedelic study, employed “supportive stance” with “no significant ACT interventions or feedback provided” during the medication phase [(10), p. 16], in spite of ACT-based approaches actively used during preparation and integration sessions. Whilst such pharmacological randomised control trials have pressures for eliminating confounding variables during the medication phase, this may sometimes be at odds with developing a psychedelic therapy if and when therapeutic context manipulation is critical to therapeutic progress (16). A more dynamic relationship between the psychedelic experience and accompanying psychotherapeutic interventions could be helpful throughout all three phases of psychedelic therapy. Recently developed Psychedelic Somatic Interactional Psychotherapy (PSIP) advocates for both directive and non-directive modalities during the medication phase, and suggest that the skill is knowing which is appropriate when [(17), p. 13]. CBS would concur that greater precision and context sensitivity is possible, and adds that a contextual awareness of therapeutic intentions and effects can clarify when and how to be directive. Flexible humanistic approaches have long had structured and directive elements [(18), p. 53, (19), p. 20] so that therapists can “make interaction happen where it isn't” [(20), p. 172]. Furthermore, context sensitive interventions during the medication phase are probably important for certain situations, i.e., if new perspectives are not experienced during the medication phase, the post medication sessions will have very little to integrate [(11), p. 98].

During the integration phase, there is also some consensus that psychological barriers such as ingrained thinking patterns, can undermine the gains of psychedelic-assisted therapy [(11), p. 98, (10), p. 13, (21)]. With data showing that half of participants relapse within 6 months [(3, 22), p. 27] a framework that addresses these barriers may be important. Unfortunately, research is lacking regarding the role of therapeutic interventions in preventing relapse.

Some important work has already been done regarding how to integrate approaches, modalities and competencies (9, 23, 24) for psychedelic therapy. This paper contributes to that thread by specifically considering what CBS can offer psychedelic-assisted therapy, how it can offer a framework for integrating different modalities already used, and how its empirically supported processes can be adapted into a new model for addressing known challenges of psychedelic-assisted therapy, applicable throughout its three phases.

### Why Is Acceptance and Commitment Therapy Appropriate to a Psychedelic Context?

As a purely psychological intervention, Acceptance and Commitment Therapy (ACT) and its model of Psychological Flexibility offers empirically supported treatments for anxiety disorders, depression, addiction, somatic health (25). Luoma et al. (24) highlight in detail the unique position of CBS to cater to many psychedelic-therapy aspects such as: (1) the crucial importance of context (16) when CBS is a study of the effects and manipulation of context, (2) accommodating spiritual and religious perspectives, widespread in psychedelic experiences (13), when these can be understood behaviourally (26), (3) a spectrum of increasingly flexible cognitive states, measured neurobiologically (27), CBS may measure and manipulate these behaviourally in terms of Psychological Flexibility (28), (5) Self-transcendence, measured routinely in Psychedelic research (29, 30) is central to the Psychological Flexibility model (31, 32). For a more detailed conceptual treatment of these overlaps and others see (24). Consiliently, a more recent empirical review of qualitative data suggests that psychedelic therapy processes include: an expanded emotional spectrum (involving acceptance), connectedness (to self and others), insights, transcendence and shifts in perspectives of self (33).

A deeper exploration of how ACT might be configured to enhance psychedelic therapy process therefore seems warranted, as these align well and especially for facilitating changes in self-perspective, a major area of research in CBS (32, 34).

### The Importance of Somatic, Non-cognitive Approaches

Another important process in psychedelic-assisted therapy is implemented with the use of somatic, non-cognitive techniques designed to access the implicit, automatic or subconscious aspects of self. There is notable critique of the dominance of cognitive, self-reflective understandings of self in cognitive neuroscience (35). Buddhist psychology has long differentiated between the continuous implicit vs. the momentary explicit. That is, an experience needs to stand out from its context before it can be perceptible: a spark in the dark is noticed, a continuous hum of a fan is not (36). Gerrans (37), highlights that much of thinking may not even be coded linguistically and that only inputs and outputs of thinking maybe explicit enough to prehended by language. Holotropic Breathwork (38), Psychedelic Somatic Interactional Psychotherapy (PSIP) (17), Focusing (39, 40), and Hakomi (41) are examples of therapies often used with psychedelics to help access non-cognitive experiences of self, inviting the implicit to become more explicit.

Whilst “second wave” cognitive approaches such Cognitive Therapy (42) have been criticised for their focus on cognitive, reflective awareness, and even attempting to manage bodily experiences such as anxiety, with cognitive responses [see (17), p. 5], “thirdwave” approaches such as ACT employ mindfulness processes to allow the body and its emotions to “speak” and open participants up to inner experiencing, rather than controlling or changing it. What is more, ACT's behavioural processes provide a framework for noticing any bodily behaviours including those that a participant may not be aware of. From a behavioural perspective, the “implicit” may be observed and even detected in terms of the behaviour that relates to it, much like how an invisible black hole in astronomy is primarily observed by the way things move around it. Involuntary bodily behaviours and sensations point to the unacknowledged or “subconscious”.

### Debating the Self Within CBS Research

**Figure 1:**
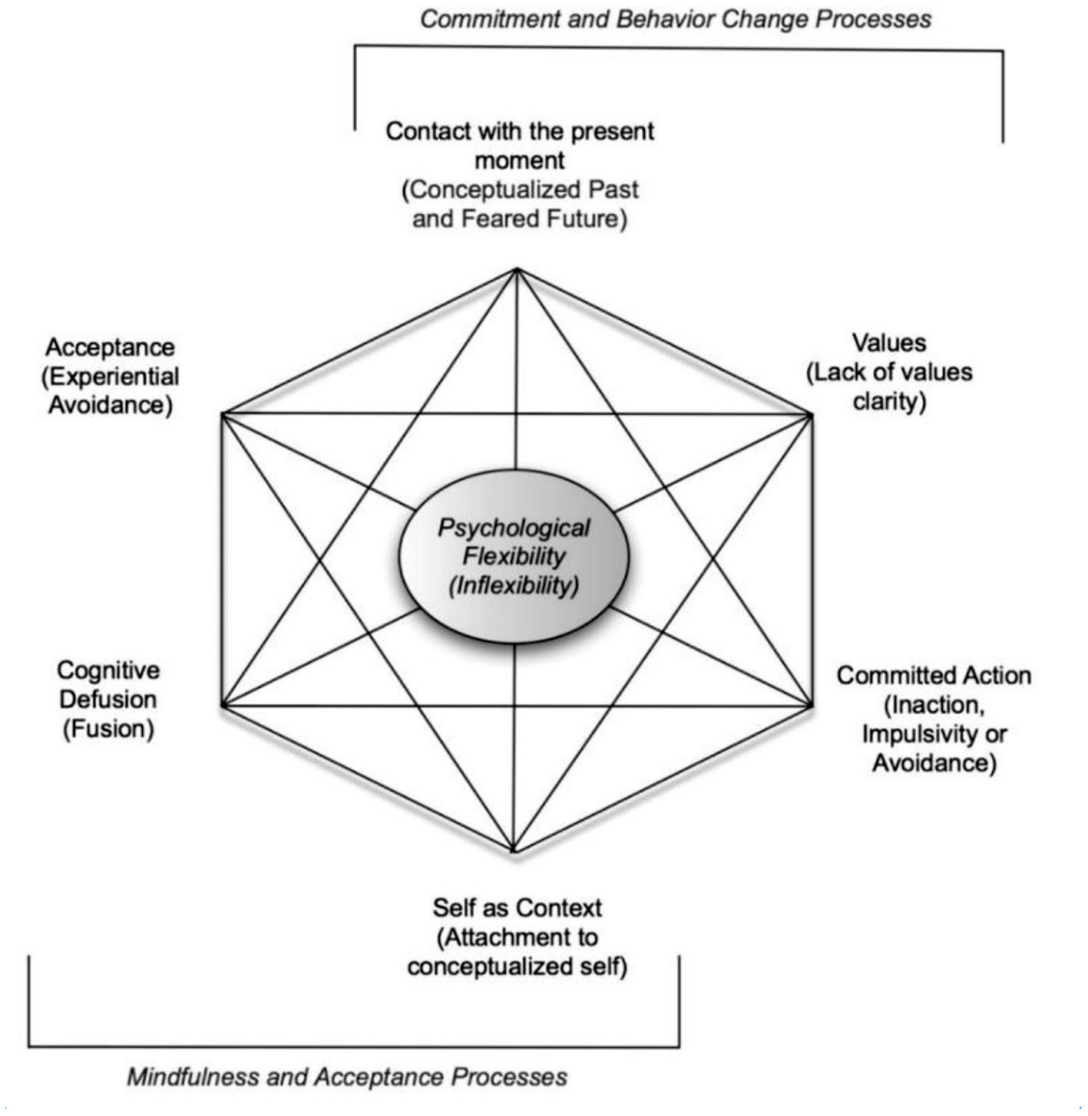
Prevedini et al. (45). Reprinted with permission.

Beyond compatibilities between ACT and Psychedelics, another factor to consider is that ACT and its model of psychological flexibility are also in a process of development, particularly regarding the Self (32, 43, 44). Even specifically in a psychedelic research context, Hayes et al. [(31), p. 35] have criticised the ACT Hexagon model (see Figure 1) for oversimplifying and underrepresenting the ACT core processes of Self.

Six processes presented as dichotomies of psychological flexibility/inflexibility. Figure 1 illustrates interrelating *core processes* of psychological flexibility as six dichotomies: (1) Contact with the present moment vs. rigid past or future attention, (2) acceptance of inner experiencing vs. avoiding such inner experience, (3) awareness of thoughts as thoughts vs. thoughts taken literally (cognitive fusion), (4) self as context vs. self as a conceptualised story, (5) committed action vs. ineffective or avoidant action, and (6) consciously chosen values vs. unclear values.

Since this dichotomous representation of the six psychological flexibility processes became widespread in ACT clinical trainings and research, more CBS research has been done into how Self processes are measured and understood. Figure 1 does not represent these newer CBS understandings of Self and neglects “self as process” [(31), p. 35]: the present moment experiencing self that interconnects the *conceptualised self* with *self-as* context, a more transpersonal and linguistically-indefinable self that contains all our experience. For the extensive empirical support for the clinical usefulness of a self as a hierarchical container, see Supplementary Material S1.1) Moving through these three selves from content, to process, to context, a person's perspective (see Table 1) is decreasingly influenced by the content it contains [(43), p. 145]. As many leading CBS researchers suggest that experiences of the self is such a central process to psychological functioning [(49), p. 5] it appears to be moving to a more central place in the ACT model (31, 32).

**Table 1:** Example of content moving therapeutically through the three selves of ACT.

**The three selves in CBS**	**Neurobiological equivalent**	**Example of corresponding cognition**
Self-as-content/story	Damasio's Autobiographical memory-based self [see (46)]	I just know I'm broken and therefore I can't love
Self-as process	Damasio's Core consciousness self (47)	In this moment, I am aware of feeling broken, and am having thoughts about my capacity to love
Self-as perspective (also described as “Self-as context”)	Less clear yet we can consider the E-Network in Legrand and Ruby (48)	I see that this “I'm broken” self doesn't help me to love the way I want to and I know I can act independently of it. My *self* that is observing is independent from and contains the self that feels broken

Furthermore, CBS researchers (24) have suggested there is a point of convergence between Psychological Flexibility and a spectrum of cognitive states known as the *Entropic Brain Theory*, suggesting different degrees of cognitive flexibility (27). This might suggest Psychological flexibility can be viewed as a spectrum of selves.

## The Psychedelic Self and Its Therapeutic Implications

Having considered the importance of the *self* in the Psychological Flexibility model (see also Supplementary Material S1.0 for more details), let us next consider psychedelic research findings relevant to variation in self-perspective:

### Experiences of Changing Sense of Identity

Qualitative analyses of participants in psychedelic-assisted therapy for cancer diagnosis distress (50) and smoking cessation (51), often experienced a vivid change in their sense of identity. An Interpretative Phenomenological Analysis, found nine out of thirteen participants experienced “Alterations to Identity During Psilocybin Experience” and six experienced “Lasting Changes to Sense of Identity” (43, p. 9). Such changes in identity increased one participant's ability to live the life she wanted (43, p. 22). A changing “sense of identity” leading to behaviour change may overlap with the transition exampled in Table 1: an older identity loses its hold over its corresponding rigid behavioural choices, allowing for new choices to be made.

### Increases in the Personality Trait of Openness to Experience

Using the NEO Personality Inventory (NEO-PI) measure of the five *factor* personality traits, MacLean et al. (52) found lasting increases in *openness to experience* after administering Psilocybin. The same measure during an MDMA for PTSD study also detected lasting openness and decreasing neuroticism (53). A cross sectional study of recreational MDMA also measured an significant increase in openness (54). This raises the question of whether this openness to experience could be harnessed for therapeutic behaviour change, and may align with the psychotherapeutic coaching that ACT provides for *opening* a person up to embracing challenging experiences [(55), p. 276, (56), *Willingness*].

### “Ego-Dissolution” —A Dissolving Self

Another well-documented concept in psychedelic research that overlaps with *self* is that of *ego*, which are terms often used interchangeably [(27), p. 2]. Many researchers have suggested that distortions in the subjective experience *self* or *ego* are “central” to the psychedelic experience [(29), p. 2, (57)]. Moreover, psychedelics can occasion a change in sense of self so radical that it appears to dissolve: perceived boundaries between self and other are lost. “Ego-dissolution” is so common that an Ego Dissolution Inventory (29) has also been validated expressly for a psychedelic neuroscience context, measuring phenomena with items such as “I experienced a disintegration of my “self” or ego,” and “All notion of self and identity dissolved away” [(29), p. 3]. Clinically we will need to consider how such an experience can help or hinder therapeutic variations in self-perspective.

Millière (58) highlights that such experiences of ego-dissolution have been documented to occur with three different types of psychedelics, each acting on different brain receptors: Classic serotonergic psychedelics, such as psilocybin, dissociative anaesthetics such as Ketamine and Kappa Opioid Receptor Agonists such as Salvia Divinorum. Another complication for the psychedelic therapist is that such experiences can be considered positive (often in terms of a mystical unity with something greater than *self*) or negative (in terms of a fearful resistance to losing *self*, or an experience of dying [(58), p. 4]. A person can experience this as traumatic and may need further psychological support to accommodate the experience.

### Mystical Experience Beyond the Self

Often an experience of a dissolving-self can lead to what is termed a *Mystical Experience*, an experience of unity perceived to be ultimate and basic to the world [(59), p. 132]. Such experiences are so well-established in psychedelics that a Mystical Experience Questionnaire (MEQ) has been validated for measuring such experiences in a psychedelic context (30). This 30 item measure explicitly monitors participants” experiences in four subscales: positive mood, transcendence of space/time, ineffability and mystical. Multiple items in the “mystical” domain relate to self. For example “Freedom from the limitations of your personal self, and “Feeling a unity or bond with what was felt to be greater than your personal self” [(30), p. 1186]. Mystical experiences may also include experiences of rebirth or perinatal memories, which are documented and explored in Grof's work (60). These mystical experiences exemplify the more extreme examples of a changing self, and can be very challenging for the participant. A psychedelic therapist will need to prepared to help patients accommodate such experiences, being open to them as they occur during medication sessions, and then making sense of them during the integration phase.

### Confusion Around a Multiplicity of “Selves”

In the context of MDMA as a treatment for PTSD, Schwartz and Mithoefer (61) measured that 68% of participants spontaneously reported experiencing different “parts” of themselves, using a non-validated measure that asked whether the participant or therapist had brought up the notion of parts. Such *parts* are often childlike or highly critical. An implication for the psychedelic therapist is whether this experience of varied selves fosters a self-perspective that can make choices independent from old self-stories. Will senses of self that are activated by emerging fears become more influential or less influential? Furthermore, multiple selves can lead to confusion and inner conflict regarding who the patient *actually is*? The absence of a familiar sense of self can be confusing. In the context of Ketamine-assisted therapy for depression, Katzman (21) calls this “…the distress that comes from the loss of a habituated identity,” with patients making comments such as “I don't even know who I am anymore, without my old buddy, Depression.” Clinically, this has implications of (1) normalising multiple *selves*, (2) which self-repertoires to reinforce or attend to, and (3) whether to address inner conflict between the varied selves that a person perceives they have.

### Inflated “Ego” and the Narcissistic Self

The Ego Dissolution Inventory (EDI) also includes a subscale for measuring Ego-inflation, employing items such as “I felt more important or special than others” and “I felt my viewpoint was worth more than other peoples”. Whilst such an inflation was much more marked in users of cocaine, an inflation was also associated with psilocybin [(29), p. 8]. Moreover, there are many anecdotal reports of narcissism being more pronounced after psychedelic experiences than would be expected, considering that the immediate effect of psychedelics is commonly a reduced sense of “ego” (rigid conceptualised self). A person can interpret intense psychedelic healing as suggesting they are somehow special or chosen [(62), p. 10]. Clinically this suggests it might be helpful to monitor a person's relationship with any emerging positive self-stories during the integration period, noticing any inflexibility around their relationship to that story. This is standard practise in ACT but it may be particularly helpful to emphasise for the psychedelic therapist, as new positive self-storys may commonly emerge during the integration phase.

### Othered Selves: Meeting Entities Which Can Be Considered Hallucinations or Actual “Sentient” Beings From Another Realm

Participants in psychedelic studies of various substances [and especially DMT (63, 64)], often report the phenomenon of meeting what appear to be autonomous “beings” [(50, 65, 66), p. (2, 67)] offering guidance or other communication. The participant may consider this guidance to come from another part of themselves [(68), p. 531]. Such entities can also be experienced as a malevolent or demonic encounter [(67, 69, 70), p. 7], and can example many Jungian archetypes, considered universal to many human cultures such as the mother, the mentor, the monster, some of which may function as a personification of the mind's dissociative defences (71). An awareness of such archetypes may help inform what content is avoided, valued or unhealthily attached to. The model below treats these common experiences as aspects of self. For a fuller treatment of how and why such entities can function as projections of self, see Winkelman (72), and for how this relates to trauma see Kalsched (73). A psychedelic-therapist will need to address such encounters and the self-perspectives they represent, whether considered real, imagined, desirable or frightening. Such perspectives will be clinically relevant to the extent they help or hinder the process i.e., they may point to neglected values, new behaviours or inner avoided experiences.

### Unresolved Trauma Informs Self-Story

It is remarkably common for traumatic memories to surface during psychedelic treatment [(74), p. 5, (60), p. 116], perhaps due to the enhanced psychological flexibility or lowering of psychological defences to enable an “unconstrained cognition” occasioned by psychedelics [(75), p. 2142]. In a study of psilocybin for treatment-resistant depression 10 out of 20 subjects attempted to avoid memories of childhood trauma, whilst eight succeeded in confronting such memories [(68), p. 525]. Note that this often happens without any explicit direction to face trauma. The recently validated *Emotional Breakthrough Inventory* (EBI) (76), designed as a state predictor for long term outcomes of psychedelic therapeutic process, employs the item “I experienced a resolution of a personal conflict/trauma” which scored highest in the factor analysis at 0.896, out of the measures' eight items, thus signifying it was the strongest predictor of *emotional breakthrough*, which predicted well-being at 2 weeks post treatment [(76), p. 14].

In trauma research, the *Centrality of Events Scale* (77) has measured how central a traumatic event is to a person's identity and life-story, identifying correlations not only with PTSD symptom severity (0.38) but also with depression (0.23), and irrespectively of whether the reported event matched the current diagnostic criteria for post-traumatic stress. This article will also extend use of the word *trauma* to include negative experiences that have a lasting effect on person's identity or self-story, and not restrict it to PTSD symptoms or life threatening events. A consilient finding in the psychedelic literature was during an MDMA-assisted therapy for PTSD study: the NEO-PI-R measured that those who had the greatest personality change following treatment (increased openness and decreased neuroticism) also had significantly greater decreases in PTSD symptoms (53). The studies above suggest facing traumatic memories can be an important component of psychedelic therapy process, and not only with PTSD cases.

A clinical implication is the possibility that such traumatic memories may not be fully processed at the time of the psychedelic medication session. Experienced psilocybin mushroom therapists have warned that participants who don't integrate their traumatic memories can actually get worse if they don't continue working therapeutically during the integration phase [(78), p. 209]. If additional support is not received at such a critical point, a patient could regard the psychedelic experience as traumatic in itself. Psychedelic treatments can access traumatic memories that were unexpected to both patient and clinician, including preverbal trauma that may be difficult to access without psychedelics [(78), p. 209]. Psychedelic therapists will therefore ideally to be prepared to help participants open up to traumatic memories during the medication phase (should they arise), and to continue trauma work during the integration phase.

In sum, these phenomena (selected due to their relevance to self-perspective) suggest a responsiveness to trauma and rapidly changing senses of self will be pertinent in psychedelic-assisted-therapy models. Furthermore, complications arising from a widely varying sense of self include: (1) confusion about one's identity, (2) narcissism or an unhelpful inflexible relationship to a positive conceptualised self, and (3) mystical experiences that can include a loss of self or unity beyond the self.

## A Psychologically Flexible Self Model, Optimised for Psychedelic-Assisted Variations of Self

The next section of this paper presents an expanded continuum of seven selves, as a psychedelic-appropriate alternative model to the six dichotomous ACT processes of psychological flexibility. The *Spectrum of Selves* model is designed to accommodate the demands of a psychedelic context, as well as increasing precision and context sensitivity of psychological flexibility processes during the three phases of psychedelic-assisted therapy.

**Figure 2:**
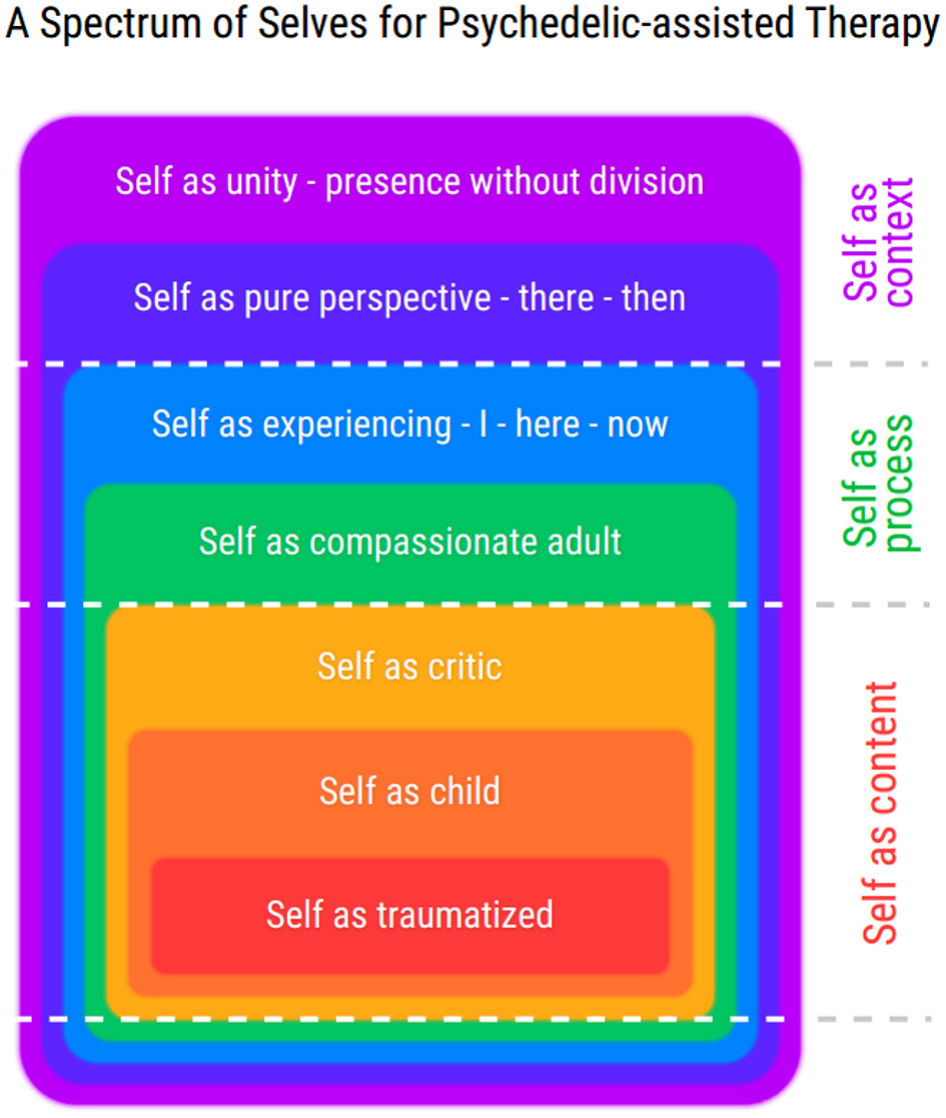
First presented at Association of Contextual Behavioural Science conference, New Orleans (Online) 2020. Reprinted with permission. These multiple selves are framed as concentric circles like Russian dolls. The varying selves are arranged according to how psychologically flexible they are. The blurred edges of each self represent the fact that self-experiences are never fully captured by language or explicit cognition, and that implicit, unseen aspects are always part of the equation. For an expanded version of this model's axes, see Table 2.

The *Spectrum of Selves* (SoS) model in Figure 2 is a new psychological flexibility model proposed to accommodate a wide range of self-experiences, as encountered in a psychedelic context, packaged as a memorable user-friendly heuristic. This accommodates a trauma-influenced self-story, multiple selves that appear to be of varied ages or attitudes, narcissistic-self stories, a compassionate self, “othered” identities such as benevolent or malevolent entities, a dissolving self and mystical experiences of unity or no self. Such a spectrum places self-perspective as the central uniting process whilst emphasising self-as-process as the first container of self-content. All permutations of the other five processes throughout the seven selves can be framed as behavioural tendencies that constitute an experience of a particular self (See Table 2). This configuration of psychological flexibility processes intends to render them easier to apply and more able to cope with the high variation in self-experience and intensity inherent in psychedelic therapy. Note that Figure 2 does not pretend to be a complete map of psychedelic experience, it simply offers helpful ways to respond to a wide range of common psychedelic experiences.

**Table 2:** Non-self processes of psychological flexibility integrated as elements of different self-perspectives, and related to psychedelic phenomena.

**Types of self: the 3 selves** **expanded to a 7 point scale** **(plotted against the other 5 core processes)**		**Cognitive** **Defusion-** **Cognitive** **Fusion**	**Values-** **Unclear values**	**Present moment-** **Automatic**	**Open acceptance-** **Closed/control**	**Committed Action-** **Avoidant action**	**Psychedelic-therapy relevance**
** 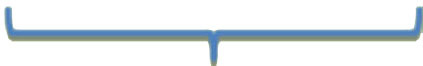 **							
SELF AS CONTEXT	As Unity	Awareness unfiltered by language.	All is love	All “is” —and beyond past and future	Surrender to all that is	Allowing transformation, through death and rebirth	Mystical Experiences to allow and make sense of
	As Pure Perspective	Language is thin and generally less interesting	Feeling what is important moment to moment	Seeing the world from beyond the habituated perspectives	Listening to all emotions as messengers	I always have the option to choose my response	New self-perspectives emerging as *Entities* (Othered selves). Challenging existential fears nearing ego-dissolution
SELF AS PROCESS	As Experiencing container	My thoughts are just thoughts/don't define me	I can choose independently of my old patterns	I see through my ways of not being present	I'm allowing myself to feel these challenging emotions	Choosing to grow through discomfort	Increased capacity for felt-sense (Somatic work)
	As Compassionate adult	I notice that's a harsh thought	I value kindness	I'm aware of what I'm doing	What does this feeling really feel like	I'm taking steps in self-care	Self-compassion and Openness to experience shame
SELF AS CONTENT	As Inner critic	I am pathetic	I must/mustn't do X to be OK.	Rigid rule following to improve sense of self	Don't get upset again	Staying busy will make me feel less	Parts work to loosen dominance
	As fearful child	Another must save me	Neediness	Automatic reactions to feeling insecure	I'm at war with these feelings	Demanding or inactively hoping to be saved	habitual/historical self behaviours (c.f. IFS)
	As Traumatised	There is something wrong with me	I only care about making it stop	Dissociation/can't be aware of it	Frozen, Can't bear to feel	Inactivity, Giving up	More accessible Traumatic memory (also addressing psychological crisis PiMS experiences)

*Many of the language examples here would be post hoc, especially for the Self-as context section where language would have lost its dominance during the experience. Such post hoc language can still be useful for recalling some sense of these experiences*.

As well as a response to Hayes' critique (discussed in Supplementary Material S2.0), psychological flexibility processes recast as scales may be more responsive to varied individuals than binary dichotomy processes as in Figure 1. An assumption here is that a scale is more precise to calibrate to an individual than a binary.

The extensive empirical support for self-as-hierarchy (detailed in Supplementary Material S1.1) also informs the model and features throughout. Selves of increasing psychological flexibility are considered to contain each other, and not as a literal fact but as a helpful way to relate to them. This hierarchical continuum of selves ranges from the most rigid fears inherent in unprocessed trauma, broadening through critical voices that have broader behavioural repertoires than a traumatised self, opening to more compassionate values-aware perspectives. At the top extreme of the continuum we experience the peak transcendental states, as measured by the EDI and MEQ, in which participants glimpse an all-containing perspective of unity, beyond any sense of separation, time, space and even language.

### Self as Traumatised

At the lower extreme of the continuum is the traumatised self. Like the *Centrality of Events scale*, this model interconnects trauma and conceptualised-self work as an important factor to consider generally. *Unresolved Trauma Informs Self-Story* (p. 5) highlighted the prominence of traumatic memories in psychedelic studies. In the most fear-ridden, traumatised states, a person may be least psychologically flexible and may be locked in involuntary dissociative responding, to knowingly or unknowingly avoid such experience. The Dissociative Experiences Scale (79) has detected such dissociative behaviour in a wide range of clinical problems [(80), p. 513], as well as non-clinical anxiety and neuroticism (81). From a CBS perspective, any behaviour can be assessed in terms of whether it is moving away from a trauma-related experience, or other aversive experience. When such aversive content is encountered, the participants opening up (or exposure) to it could be encouraged whether during the preparation, medication, or integration phase. As discussed in *Unresolved Trauma Informs Self-Story*, it may be important to assess whether any traumatic material contacted has been integrated, or if it needs further attention via a trauma-focussed exposure therapy during phase three.

### Self as Child—Self as Critic

*Confusion Around a Multiplicity of “Selves”* (p. 5) highlighted the prominence of a multiplicity of selves often emerging in a psychedelic context, and the challenges and confusion that can result. Many different experiences of *self* can potentially emerge, yet a child and critic protector seem to be particularly common. Internal Family Systems (IFS), a psychotherapy commonly used with psychedelics (especially MDMA), offers perspectives and therapeutic tasks, to highlight common functions and relationships between such parts (82). Validating and normalising the motivations of these *usual suspects* can facilitate a self-compassion and more nuanced perspective of self-as-content-driven behaviours. This serves cognitive defusion, acceptance and enables more choice beyond old habits. The SoS model places such *parts work* within a CBS framework of hierarchical, containing perspectives with varied degrees of distance from psychological content and/or varying degrees of psychological flexibility. The purpose of this integration of approaches is that the nuanced empirical support of CBS self-processes may inform what elements of IFS and ACT are most important.

ACT therapists are already very familiar with inner “parts” or “voices”, and use a metaphor of *bus passengers* who are distinctly not drivers (active choice makers) of the person's *bus* (chosen life behaviours) [(55), p. 250]. An extension of this with Internal Family Systems (IFS) highlights common functions and relationships between the bus passengers or *parts*, in order to enable a deeper acceptance, more self-compassion and less conflict between the *parts*. Dialogue with and acceptance of such *parts* may also help access key content such as shame or traumatic memories.

If an inner critic *contains* the child, it does so in the sense that it *sees* the child's behaviours as optional, and suggests alternatives. If the child (or critic) *contains* a trauma it is in the sense of a self-concept wound, around which some of its personality serves to deny or make up for. The intervention of having a person discern and respond to younger child parts, has some empirical support for treating borderline personality disorder in the form of Schema Therapy (83), with outcomes of decreased depression, reduction in early maladaptive schemas, and increased quality of life. *Parts work* also has some support in the IFS data for an MDMA-assisted treatment for post-traumatic stress [((61), 71), p. 257], with outcomes of reducing PTSD symptoms and increasing openness to experience (53). IFS parts work without psychedelics has some promising empirical support for treating Rheumatoid Arthritis (84) and depression (85). Van der Kolk [(86), p. 278] concurs on a connexion between trauma and warring parts of self, and suggests that exploring and befriending such parts is an important component of healing.

In sum, these categories of Trauma, Child and Critic help to explore *self-as-content* from multiple self-perspectives. Participants and clinicians can consider which contextual cues invite these different *parts* to act out. Any number of inner parts (including entities) can be assessed in terms of the degree they influence the person's behaviour and any new perspectives they offer about that person's life that can inform the therapy. For an *entity* as *part* example see *Malevolent Parts* in Supplementary Material 3.1).

### Self as Compassionate Adult

Containing the self-trauma-wound, the child and the critic, a person might next assume a *Compassionate Adult perspective*. Stepping into Self-as-Process, and bridging towards self-as-perspective, this mode of self is aware of the ever changing momentary impulses of the conceptualised selves without automatically acting on them. This compassionate part and is more in contact with a longer term view of a life and is more capable of values-driven behaviour (rather than avoidance-driven). It compassionately contains self-story with more metaphorical *distance*, and without taking it so literally. It is may also use self-story healthily: i.e., *I am someone who likes to be kind*, In addition, this awareness and acceptance of less flexible modes of self, not only brings self-perspective-taking into the heart of an ACT-consistent model, but also make self-compassion a centre-piece. To see our warring impulses as upset younger *parts* who just want their needs met is helpful in bringing acceptance at the level of identity. Active self-soothing and self-care can be formulated as the acceptance of specific emotional *parts* with particular perspectives. For some time the lack of explicit focus on self-compassion in ACT processes has been questioned. Luoma and Platt (87) suggest that a greater emphasis on self-compassion during ACT work may increase effect sizes, especially when self-stigma and shame are relevant across many diagnostic categories.

### Self-As-Experiencer—I Here Now

Less identified with any particular view of self, the self-as-experiencer holds the compassionate adult lightly and keeps track of when compassionate and positive self-story behaviour is appropriate, inwardly and outwardly. In certain contexts it will be appropriate to let the child cry, or the stern critic to hold their ground with appropriate anger. Any new (positive or negative) sense of self can easily lock into a new inflexible story with a new set of trappings e.g., “I am a good compassionate person so I must never express anger.” This mode of self is more aware of the filters of cognitive evaluations and maintains awareness of the function of any *part* or any *self-story*. It is also increasingly open to experiencing bodily feeling, without recourse to cognitive descriptions. The experience of an “I” looking from a particular here and now is still very present.

### Self as Pure Perspective

As psychological flexibility increases further still and the verbal/cognitive influence of the mind-brain over present moment experiencing reduces accordingly, a sense of self-as-perspective is more easily accessed. That is, anything the person does, sees, feels is only a part of the perspective and ultimately does not define who they are. The sense of “*I”* softens. Cognitive-emotive content is experienced more as *there – then*. Whether achieved pharmacologically, though meditative practise or therapeutic intervention, much greater shifts in perspective are possible which may reduce the overriding dominance of habitual perspectives. This greater capacity for self-perspective may enable radically new views of past, present and future containing new possibilities not previously conceived, as well as increasing the ability to understand the perspectives of other people, and even Nature as a collective. Entities and archetypes are also more likely to be experienced as the brain's top down control of reality becomes less rigid, and the habitually supressed parts of a person's psyche are allowed to emerge in one form or another. Like the inner critic and child these can also be responded to with parts work, as they may also have protective functions. For example a threatening God is an archetype that can embody both malevolent and benevolent protection at once. When such protective functions are recognised and validated other important content such as traumatic memories may become more accessible [Kalsched (73) in Hill (71)].

### Self as Unity

At the top of Self-as-Context (the hierarchical containing self) and the top extreme of the spectrum, we find Self as Unity: the ultimate all-containing perspective in which experience is least influenced by syntactic language. As detailed in section Mystical Experience Beyond the Self, peak mystical experiences are an important component of psychedelic-assisted therapy. An experience of ego-dissolution (see section “Ego-Dissolution” —a Dissolving Self) may be a close precursor to what participants describe as an experience of absolute unity in which there is no time, space or division of any kind. This suggests that the *I-You, here-there and now-then* relational frames of syntactic language have largely dissolved. Whilst participants may only remember non-verbal ineffable aspects of such an experience, the journey there and back may offer a new experience regarding the psychological flexibility possible for a person's self-repertoires in emotive (acceptance/openness to feeling), spatial (here-there) and temporal (now-then) experience, as the influence of syntactic language fades and returns. During mystical experiences that may closely precede or follow moments of unity, *entities* offering new perspectives on the person's life may also occur. After the experience it may be helpful to try and encode something of what happened in syntactic language to bookmark any new self perspectives attained that could inform how the person wishes to live their life going forward.

fMRI imaging has already detected increases in brain activity in both spatial and temporal domains (88), suggesting changes in here-there and now-then perceptions occur even at medium doses (75 μg of LSD).

Clinical implications will include assessing whether such an experience could be too destabilising for some populations, and when lower doses that are less disruptive to a person's sense of self may be more appropriate. These are still helpful for expanding awareness of the autobiographical self-as-content but without experiencing the challenges of unity and the narcissistic ways the self-as-story parts might respond (see section Inflated “Ego” and the Narcissistic Self). Also, irrespective of whether a mystical unity was experienced as something positive or negative, it is likely be fruitful to revisit and unpack such experiences. Doing so may help a participant appreciate different perspectives of self that in turn could increase experiential contact with neglected values or fears that are important to address during the integration phase.

Table 2 Illustrates in more detail how the five non-self core processes can be mapped as axes of different selves across the spectrum. The exercise of tracking any thought, emotion or behavioural responding as belonging to a particular self-repertoire, serves to foster awareness of self-perspective throughout a person's experience. Note that they are generic examples in Table 2, and are not to be taken as universal. These five spectra are **not** intended as a measure of where a person is at, but offered as a process that fosters perspective. For example a person could have a feeling of “worthlessness”, notice that the feeling has a long history with a child part and choose a valued behaviour in spite of the feeling, from a compassionate self-perspective. A person can be in touch with two or more self-perspectives at once. Conversely a person could feel they are “one with the universe” and destroy things that matter to them, unawares. In such a situation the feeling of oneness could be assessed as having the function of bypassing other feelings, and may be recast as a conceptualised higher-self that the child or critic inflexibly identifies with [c.f. *Spiritual Bypass* in (9), p. 10]. Parts work extends a containing perspective for accommodating multiple selves, whether that *part* is a recognisable historical self, or a seemingly foreign or *alien* archetype.

### Finding Hidden Barriers to Growth

This heuristic “spectrum” can be presented like a the notes of a musical scale to explore up and down, without defence or bias. Chasing positive “high” notes and avoiding negative “low” notes can be addressed with the *reverse compass* metaphor (89). That is, what do thoughts, feelings, personality structures tell a person to do or not do? If there is an urge to not feel or experience something, then this “advice” can be *reversed* as advice to feel something. If an inner part is particularly attached to having a positive experience, again the “advice” can be reversed to allow experience to unfold as it is, remaining in the present. This approaches aligns with a classic psychedelic therapy technique of *in and through* in which a participant is instructed to look a demon in the eye [(13), p. 86], rather than to avoid it because it is fear-provoking. The *reverse compass* expands this to all behaviour including subtle thought or bodily tensions that can block feeling with stealth. Thus, a path of noticing increasingly rigid selves/behaviours could also signpost a way to more deeply avoided inner experiences such as trauma-related content or fear-preceded opportunities for *Pivotal Mental States* [*PiMS*; (90)]—transient hyper-plastic states of the mind-brain that may be important for deeper and more lasting therapeutic progress. Encouraging an awareness of the function of any mode of self may help to increase psychological flexibility throughout the spectrum. For example, with permission a clinician could ask “Is there something that critical part of you doesn't want you to feel?” or somatically, “What do you feel in your body?—Is that tension in your belly trying to stop something?—What is that?”

In sum, this spectrum of selves both addresses recognised shortcomings in the dichotomous ACT Hexagon model (see Supplementary Materials S1.0–S2.0) and is prepared for a wide range of psychedelic phenomena [see *The Psychedelic Self and it's Therapeutic Implications* (pp. 3–6)]. That is, it is configured to (1) assess for and respond to self-story events that contain traumatic (memories), (2) normalise and respond to multiple selves, (3) place self-perspective taking as the central process (4) is ready to track the function of any new self-stories emerging after a psychedelic experience, (5) places compassion as a central response to variation in self, and (6) is prepared for altered-states of consciousness. For further applications of the SoS model (see Supplementary Material 3.0–3.2).

Having considered a spectrum of selves for facilitating psychological flexibility during psychedelic-assisted therapy phenomena, this article will next address how to sustain behaviour change that has coincided with temporary increases in neuroplasticity, and may be harder to maintain over time.

## Reinforcing Behaviour Change Systemically In Psychedelic Context

“As one individual changes, the system changes.” – **Ram Dass**

*Consensus and Debate in Psychedelic-Assisted Therapy* (p. 2) highlighted the issue that half of study participants often relapse within 6 months. This could be a reflection that variability in neurobiology and behaviour is not enough for stable change and that methods for retention of new behaviour are important [(24), p. 143, (91)]. How might newly emergent self-perspectives be reinforced or retained in a psychedelic-therapy context? On leaving the therapeutic container of a psychedelic-assisted session, a participant may have gained new self-perspectives and have greater psychological flexibility with neurobiological support, and yet returns to a life containing contexts that likely reinforce pre-treatment self-perspectives. Unless the new perspectives and corresponding behaviour are reinforced, the benefits may be short-lived. Ram Dass' quote above may seem doubtable when a system resists that change. How therefore might new self-perspectives and their corresponding new behaviours be successfully reinforced or even amplified during integration?

### A Temporary Window of Acute Psychological Flexibility

There is preclinical neurobiological evidence for increased neuroplasticity following medication of psilocybin (92). This also has some clinical neuroimaging support (33) that suggest a dynamic and neuroplastic process can be initiated by psilocybin, then sustained for at least a number of weeks before rebounding [(33), p. 8]. A cross sectional study also links psychological flexibility as a mediator of the acute effects of psychedelics (93). In addition, clinical opinion [(94), p. 243, (11), p. 98, (78), p. 253] also suggests that the timing of the integration phase can be optimised to take advantage of a temporary “afterglow”, during which psychotherapeutic interventions maybe more impactful. Clinically this raises the question of how the pharmacological benefits of the psychedelic “afterglow” phase might be extended or deepened psychotherapeutically. As well as aligning a conceptual model to respond to the phenomena of psychedelic experience (as suggested above), these data suggest interventions for reinforcing new behavioural repertoires be administered in a timely fashion—can one intervene too soon or too late? Hypothetically, varying, selecting and reinforcing new behaviour when it is easier to do so may reduce relapse and even realise new therapeutic potentials that may not have been accessed without integration therapy. The following behaviour reinforcement model is therefore designed to be applicable within a short timeframe.

### Barriers to Sustaining Rapid Psychedelic-Assisted Behaviour Change

A recent meta-analysis concluded that psychedelic-assisted psychotherapy effect sizes were greater than known psychotherapy or pharmacological interventions to date for: PTSD, anxiety/depression associated with a life-threatening illness, unipolar depression, and social anxiety among autistic adults (95). A greater increase in psychological flexibility (if corresponding to these larger effect sizes) may invite a higher degree of behaviour change. This assumes a new self-perspective that can make choices more independently of old cognitive content may potentially make a greater number of new choices during the *afterglow*, compared to smaller effect size therapies. Changes in behaviour are anecdotally so pronounced after psychedelics that retreat centres routinely advise their participants during integration, not to make radical decisions too quickly [(62), p. 171]. However, a person's old circumstances may not support (or even discourage) the behaviour change that they wish to sustain. In proportion to this increased psychological flexibility, higher degrees of behaviour change may be met with more systemic barriers to sustaining that change. For example, what if a participant's partner is disapproving when their partner engages in new projects, disrupting a previously comfortable system?

This article will consider how to adapt contextual behavioural reinforcement to respond to any complications arising from larger degrees of self-variation, corresponding systemic barriers to such behaviour change, within a temporary window of increased psychological flexibility.

A more systemic “Extended Evolutionary Meta-Model” of processes of change, has already emerged in CBS (31) integrating the evolutionary meta-processes of variation, selection and retention through multiple levels of behaviour: physiological, social and cultural. That is, behaviour change can be understood as a three stage process beginning with variation, instances of which are selected for their helpfulness and are retained by some form of reinforcement (96). The multilevel perspective is an extension of the view that context influences behaviour, in that most human behaviour is influenced by the relationships and sociocultural contingencies that surround that individual. For example, the frequency and type of dissociation a person experiences is very likely influenced by how that person's culture relates to dissociative episodes (97): what one culture pathologizes as “psychotic” another could value as “spiritual”. Either of these responses could increase or decrease psychological flexibility depending on the context. The approach described in this article will also consider what systemic, sociocultural barriers to change may need to be addressed, as well as what social opportunities may be harnessed to support ongoing behaviour change?

In psychedelic literature the importance of socio-cultural support, or community during integration is commonly emphasised [(62), p. 167, (78), p. 233]. There is even data that the intersubjective experience of a psychedelic therapy community predicts enduring changes in psychological well-being and social connectedness (98). Let us next consider how to systematically foster helpful behaviour change on multiple levels of cooperation.

### Reinforcing New Self Repertoires Through Multiple Levels of Cooperation

Bourzat and Hunter (78) routinely explore and encourage change broadly, through four domains of (1) Self-care (2) Relationships (4) Community, and (5) Environment. Note that these also contain each other in concentric circles and provide multiple perspectives for a more penetrating view of a person's world. Such a concentric and hierarchical view of life is far from new and has even been called a “fundamental structural principle” [Jacobson in Wilber (99), p. 57], and has previously been used for coaching behaviour change [(100), p. 245]. The needs of an individual, a relationship, a community are often different and yet interact. The immediate and natural environment also contain and influence the other three levels. For new life choices to be sustainable long-term it will be helpful if the various interacting parts of an individual's life are not in conflict with each other (e.g., an abusive relationship that prevents self-care). The bigger the change, the more rearranging of the parts of a person's life may be necessary. Any lasting behaviour change will ultimately influence situations in more than one of these domains. It can be a considerable undertaking to keep track of the new self-perspectives and the barriers they meet. Some useful perspectives may otherwise be forgotten, or actively neglected if they conflict with other forces in a person's life.

**Figure 3:**
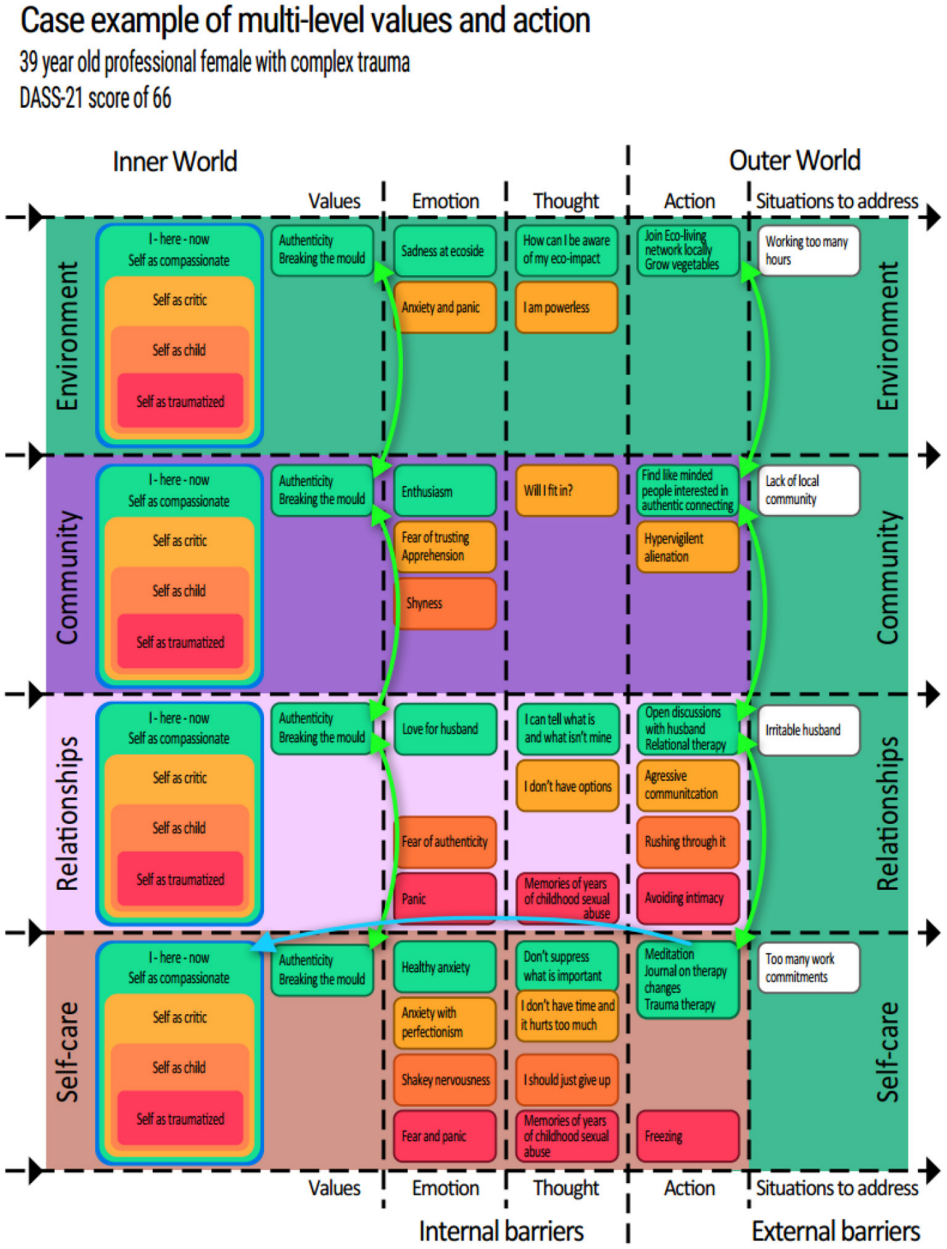
First presented at the European Behavioural Pharmacology Society preconference Workshop, 2021. Reprinted with permission.

The multilevel grid (Figure 3) offers a framework for detecting and tracking any barriers to sustaining change, as well as interconnecting motivations and effects that reinforce such change. Figure 3 displays an anonymized example of a complex trauma case with moderate depression and severe anxiety (DASS-21 score of 66). Such a visual tool can help both participant and clinician to keep track of and connect new perspectives to new behaviours. Its purpose is also to identify systemic barriers to change and find self-sustaining behavioural paths to long term change.

### Coherence as a Reinforcer for Change

Coherence is a powerful reinforcer and incoherence, a powerful punisher [(32), p. 106]. The power of a functional coherence therefore could be harnessed to cultivate contexts that reinforce the desired behaviours. Any incoherence can be assessed as to whether it punishes the desired behaviours. The participant detailed in Figure 3 received four integration session during which the content of Figure 3 was collected and formulated. This visual representation tracks the flow of behaviour change from inner self-perspectives to outer world interactions across personal, relational, communal and environmental situations. The green boxes, represent inner to outer behaviours/events that this participant wished to cultivate through the integration phase: moving through potential inner barriers of emotions and thoughts (e.g., the yellow “*I don't have time and it hurts too much*” may try to block the green selfcare practises such as “*Meditation”*), to outer behaviours and the outside situations these behaviours will meet and hopefully influence (far right). The red, orange and yellow boxes map behavioural paths of self-as-story patterns that may have become clearer during the psychedelic medication or integration sessions. This is not to label them as bad, but to increase awareness, acceptance and choice around them. Supplementary Material 3.2 highlights how even a rigid narcissistic self can attain a healthy effect in the right context.

As well as mapping behaviours from left to right, it also allows for mapping behavioural coherence between levels (up and down). For example a small daily act such as meditation (see Figure 3, bottom left) may be *augmented* with the appetitive functions of strengthening helpful behaviours (both inwardly and outwardly) across the four domains. This meditation can serve maintaining a compassionate self-perspective with increased psychological flexibility and compassion towards a partner, increasing openness to community involvement, thus contributing to motivations, reinforcers and desired effects with a single behaviour. Thus, reinforcement can come from multiple domains of a person's life.

Conversely this approach also suggests we routinely look for behaviours out of alignment with a person's most important values across these four domains as well as reinforcing behaviours that increase harmony between domains. Relationally, community members could support an individual's self-care and vice versa. The curved green bidirectional arrows represent how such valued action on one level could align with and contribute to valued action on another level. Whilst behaviours and values do not technically “conflict” in that it is often appropriate to make different choices in different contexts, competing behaviours (e.g., smoking vs. not smoking) can be out of balance, be experienced as incoherent, and use up limited resources that could be used elsewhere. A consciously chosen cross-domain functional coherence can increase a person's awareness of reinforcing contexts to cultivate, and reduce the effects of punishing contexts that need to be addressed. Such a detailed, coherent perspective is intended to be more robust in times of stress, post treatment. If situations that challenge a person's therapeutic progress are addressed or pre-empted, stressors maybe better accommodated and long term change more likely. An example of a systemic *incoherence* to address in a treatment plan could be: an abusive relationship and an absence of community. The abusive relationship would likely counteract self-care practises. An addition of community could help reinforce self-care. Typical ACT interventions would then suggest an exploration of new relational values, consider a critical decision such as leaving a relationship and contemplate options for connecting to a community.

In this case example, a woman with a history of years of childhood sexual abuse contributing to hypervigilance and difficulties with relational boundaries, saw that the value of *breaking the mould* could inform behaviour throughout all four domains, and thus she had values to unite her life simply. Responses to the “situations to address” (far right) could be prepared and related to the emergent new self-perspectives (far left), including increased acceptance of when older (self-as-content) selves re-emerge in times of stress.

### Cultivating Contexts to Reinforce New Selves

In addition, note that the blue arrow from the bottom right green box (“Meditation, journal on therapy changes, Do trauma therapy) loops back to the Selfcare “Self as compassionate adult” (far left). This is to highlight another intended function of the newly emerging behaviours. Not only do behaviours influence the outside world, they may also influence the person's inner sense of self. This bottom right green (Compassionate adult—Self-care) box may be of particular importance if it can have the multiple functions of: (1) Increasing the ability to remain present and potentially extend the afterglow, (2) Increase the ability to follow the (green) compassionate adult pathways throughout all four domains, which (3) increases likelihood of addressing the situations (white boxes—right side) to the extent that compassionate self-as-perspective behaviour can be readied for these and therefore become more habitual. Such coherence and multiple functions of behaviour aim to cultivate a new context (or life situation) that continues to promote desired behaviours long term. The change process can become self-reinforcing.

### Action Implications

This grid can either guide case formulation with a supervisor or be actively shared with the participant as an interactive spreadsheet. A visual of increasing alignment and coherence in a person's life can be motivating. However, this plan for a coherent multilevel reinforcement for behaviour is not a replacement for more somatic or experiential work that can also serve integration. The participant in Figure 3 spent most of her integration sessions engaged in intense imaginal re-experiencing of early childhood events, and emotional conversations with inner parts. Thus, her integration therapy enabled further exploration of the implicit felt-sense in her body, a process seemingly enhanced by the increased psychological flexibility (measured on a daily basis with the *Brief Acceptance Measure* (101). This visual reframing of her new and old behaviours may have increased her willingness to face traumatic memories, a process that could in turn help maintain her capacity for self-compassion. The checklist in Supplementary Material 4.0, offers example questions of how the principles behind this grid can be operationalised in practise. See also *Supplementary Multilevel Integration of Self-perspectives Worksheet* for an editable version of this grid.

In summary, section *A Temporary Window of Acute Psychological Flexibility* emphasised the relevance of timing of the integration phase of the therapy. Then sections: *Barriers to sustaining rapid psychedelic-assisted behaviour change, Reinforcing new self-repertoires through multiple levels of cooperation, Coherence as a reinforcer for change, Cultivating contexts to reinforce new selves, Action Implications* explored practicalities of increasing and maintaining behavioural reinforcement.

Enacting and reinforcing such behaviour changes when neuroplasticity and psychological flexibility are heightened, could be considered as a metaphorical *striking while the iron is hot*. If the psychedelic compound metaphorically *heats* the *iron*, new behaviours and their reinforcers can intentionally *shape the iron* in the light of the participant's new perspectives (before the iron *cools)*. If the new habits then achieve a contextual change that provides new contingencies to retain the new self-perspective repertoires, relapse may be less common. Thus, pharmacological and behavioural approaches can work more closely together.

## Discussion

This paper has considered how to adapt recent contextual behavioural science research to the challenges and particularities of psychedelic-assisted psychotherapy, hopefully clarifying a middle ground between the two fields. The approach presented attempts an integration not only in the sense of assimilating and consolidating the perspectives gained from a psychedelic journey, but also in terms of living a better integrated life in which the many parts of a person's life can be more in harmony with each other, both inwardly and outwardly. An assumption is that larger therapeutic changes may sometimes require such a multi-level integration to be sustainable, and those that do not require it can still benefit. This model also aims to dovetail with both ends of a psychedelic experience in view of deepening and extending the therapeutic processes as seamlessly and effectively as possible. For example shame work during the preparation phase could facilitate access to more challenging self-as-traumatised content during the other two phases. Section Action Implications also gave the example that experiential exposure work might be enhanced during the afterglow phase. The integration phase could therefore also be about accessing new implicit therapeutic thresholds that were inaccessible previously and thus maximising efficiency of the therapy before the opportunity is lost. In sum, each therapy phase forms part of the context for the next, and so a deeper preparation could contribute to a deeper therapeutic process during the medication phase, which can be continued and consolidated during the integration phase systemically. For a visual summary of these phases see Supplementary Figure 4.

The introduction to this paper touched on the debate regarding whether, when or not it is appropriate to be directive, and how to do so whilst respecting the participants experience. This model may make such directive decisions easier, if the participant is acquainted with their *inner critic* (see section Self as Child—Self as Critic) and *reverse compass* (see section Finding Hidden Barriers to Growth) during the preparation phase. A functional awareness of inner parts/urges in terms of whether they move a person towards a value/personal growth or away from a pain, can be part of the participant's mind- “set” as the psychedelic medication session begins. If a participant that is stuck in a critical attitude towards his/her psychedelic experience, they could be “unhooked” (cognitively defused) from their attitude with the simple question, “Might that be your inner critic?”. This has been used successfully in this way by Lucyne Pearson (personal communication, 2020). Such an intervention could be important during the medication session when a lack of progress at that point may prevent a participant reaching the integration phase. Ways of facilitating an openness to new self-perspectives and any barriers to this are likely to be useful throughout the three phases. During the (often costly) medication session there is a brief and critical window of opportunity which otherwise might be missed.

The functional view of positive self-perspectives (that may become new self-stories; see sections Inflated “Ego” and the Narcissistic Self, Self-as-Experiencer and Supplementary Material S3.2 *Healthy Subclinical Narcissism*, for examples) could also contribute to the debate in psychedelic therapy research regarding whether acute positive mystical experience is a key mediator of long-term success in psychedelic therapy (102), or conversely might willingness to face emotionally difficult feelings/trauma be essential to therapeutic progress? (76). Agin-Liebes et al. (6) suggest that mystical experiences may be less important according to their long term follow up data for cancer-related distress. Richards [(103), pp. 329–330] suggests either extreme can help to access the other. Carbonaro et al. (104), in a survey of 1,339 people who experienced “challenging” experiences with psilocybin found that 84% endorsed benefitting from their “bad trip” and that difficulty of experience was positively associated with enduring increases in well-being. Through a functional analytic lens it seems likely that either end of the continuum can increase or decrease psychological flexibility in different ways, and that is it not the form of the experience (mystical or challenging) that predicts its effect, but whether or not a person relates to it with an ongoing awareness of self-perspective and function. The necessity of facing difficult inner experiencing is considered pivotal in the majority of psychotherapies and it is unlikely that psychodynamic issues of the conceptualised self will fully resolve without addressing the content that maintains them.

Another question this *Spectrum of Selves* model raises is whether psychedelics will facilitate a broader view of psychological flexibility. Is it possible to be too psychologically flexible? i.e., to operate one's body (during a mystical experience for instance), or is it unhelpful to consider the mystical experience as an extreme of psychological flexibility? Heuschkel and Kuypers [(105), p. 15] highlight that the effect of psilocybin is not unidimensional: whilst it improves openness to feeling, it also impairs executive functioning such as control over thoughts, which may be therapeutically useful, but unhelpful for manual tasks such as driving a car. This suggests that different dimensions of psychological flexibility could relate to different faculties of neurobiological functioning, and that investigating these could inform a more nuanced understanding of variation in *psychological flexibility*. Optimum degrees of such flexibilities could be determined for individuals, perhaps in the service of enabling better support through pivotal mental states (PiMS). Thus, psychedelic neuroscience could inform definitions of psychological flexibility in CBS. Earlier section Why Is Acceptance and Commitment Therapy Appropriate to a Psychedelic Context? (p. 2) touched on Entropic Brain Theory that suggests a spectrum of cognitive flexibility has different properties at different points, like *criticality* in complex systems, the combined effect of multiple forces reach a balance that enables distinct properties, only at particular points in the spectrum. This could potentially inform future studies that attempt to discern the less common, yet therapeutically useful properties contained in a fuller spectrum of psychological flexibility.

There are many therapeutic modalities that can aid the development of psychedelic-assisted therapy and this article has just touched on some of them. Perhaps the unique offering of CBS is its package of precise lab-tested definitions of self, its understanding of how context influences and sustains behaviour change (essential for lasting integration) and its functional analytic framework for integrating therapeutic modalities, not based on their *form* (their traits and attributes) but on their *function* or actual effects in context moment to moment. For example the *form* of labelling inner experiencing with cognitive reflective language could have the effect of taking a person away from their on-going inner experience as they take refuge in a new abstraction, **or** such labelling could be used as a shortcut to experientially reactivate that specific inner experience [(106), p. 105, (39)] thus opening the door to new behaviours that could be selected and retained. Thus, cognitive and somatic approaches may work hand in hand, perhaps united in the task of enabling helpful variation and subsequent selection of new behaviours that lead to a more consciously chosen life.

Finally, even though ACT core processes already include mindfulness processes to open up to experiencing emotion in the body, the author recommends CBS research and interventions consider more explicitly how somatic approaches can functionally deepen *self-as-process* work.

## Limitations

The objective of this article is to set a coherent theoretical foundation for a CBS-consistent psychedelic-assisted therapy, that draws from process data and theory from both CBS and psychedelic research. However, whilst the author attempted a thorough review of the literature, it is not feasible for it to be exhaustive, particularly regarding models of self, one of the oldest subjects of study known. It is also normal that *cherry-picking* will have occurred in favour of data and models that align with CBS principles. This functional model invites further revisions as more critique is received. It is very likely the seven modes of self (see pp. 6–9) can be better defined and more closely aligned with neurobiology, Relational Frame Theory (See Supplementary Material 1.0) and mystical experiences of self [see (107)]. It is also beyond the scope of this paper to treat in detail all the relevant neuroscience that could inform this model, and the author particularly invites such critique that could improve our understanding of psychological flexibility mechanisms.

Finally, the new approach here described has only been test run and developed in a Psilocybin context with most participants without a formally diagnosed clinical disorder. The process and outcome data collected are still under evaluation. As different psychedelic compounds have different effects it is also likely to be less helpful with psychedelics that have a significantly different neuroreceptor profile such as Ketamine. However, it can function as a CBS-consistent psychedelic-optimised starting point for psilocybin-assisted psychotherapy.

## Future Directions

Longitudinal, high-density idionomic series data could help clarify how different elements of this model perform in different contexts and populations, and not only tailored to DSM categories but also to individual antecedents and consequences of presenting symptoms. Group Iterative Multiple Model Estimation [GIMME—(108)] analysis of such data could help to differentiate how changes in psychological flexibility occur differently for different people in different contexts, with different mind- “sets,” at different stages of psychedelic therapy, finding subgroup processes based on functional data rather than the form of their symptoms. Such data collection could be coupled with randomised double-blind placebo-controlled trials in which the different elements of this model are isolated, to investigate specific hypotheses such as:

1. Do new behaviours that cultivate increased coherence and alignment between personal, relational and communal domains predict lasting changes in behaviour and well-being? If so, with what clinical populations/subgroups is this most necessary or helpful? Participants at 6 months follow up could have their behaviour change examined with such a multilevel grid to see if and how behaviour change was reinforced, and whether systemic behaviour change negatively correlates with relapse.
2. Do preparative self-perspective taking exercises during the preparation phase increase the extent of perspective taking during the psychedelic experience? If so, do these in turn lead to more opportunities for behaviour change and better outcomes during the integration phase? This could be done with daily process measures throughout the three phases, seeing if self-perspective, in one phase predicts self-perspective in the next phase, and whether this ultimately predicts behaviour change post therapy.
3. What self-perspective exercises could help those who struggle with the psychedelic experience during medication session? Simple perspective taking exercises or lack thereof could be applied to different cohorts/conditions, with participant permission and titrated directiveness.
4. Does psilocybin increase the effectiveness of trauma-focussed imaginal exposure during the afterglow period? Such a study could focus on a population with a trauma history. Measures of process and outcome could collect data to compare between randomly assigned conditions that receive imaginal exposure and at different time points. This might elucidate whether and when new thresholds of therapeutic possibility are available.
5. Does a trauma-ready model increase access of aversive memories during the medication session and/or integration phases? If so, does this improve outcomes for non-PTSD symptoms such as anxiety and depression?
6. Does the addition of extended parts work to self-as-content improve therapeutic outcomes (vs. the standard ACT Bus metaphor)? This could be examined with randomised conditions.
7. Does a tracking of the function of positive self-stories during integration reduce narcissism, as measured by the Ego-dissolution Inventory (EDI)?
8. When exercising varied self-perspectives, to what degree (intensity and duration) can Psychological Flexibility be increased and extended during the integration phase of psychedelic-assisted therapy? This could be examined with a multiple-baseline approach.
9. Does variation in the three (or seven) different selves during the therapeutic process, followed by selection and retention of new self-repertoires predict outcomes? Measures such as the *3-dimentional Reno Inventory of Self Perspective* (*3d-RISP)* (109) are already demonstrating the three selves can be measured for such a purpose. Qualitative interviewing of the participants could also capture data regarding whether such processes were helpful for initiating and sustaining therapeutic gains.
10. Does somatic work (such is in a modalities like PSIP or Focusing) applicable in all three phases, improve outcomes. This could be measured quantitively, qualitatively and longitudinally throughout the three phases to determine how and when this is useful.

## Conclusion

This article aimed to integrate psychedelic science and contextual behavioural science (CBS), and in a way that can inform therapeutic interventions for multiple psychedelic therapy challenges. This trans-diagnostic, Spectrum of Selves (SoS) model offers a workable approach for fostering variation in self-perspective throughout the three phases of psychedelic-assisted therapy. Such change can be reinforced systemically with a functional coherence across multiple domains of life, in the hope of preventing the common problem of relapse. A strength is that it aligns with a range of psychedelic phenomenological data, RFT hierarchical responding research, contextual behavioural reinforcement data (important for sustaining benefits), archetypes that personify the mind's dissociative defences, parts work and psychedelic neuroscience reviews such as Entropic Brain Theory. A weakness is that it is just the beginning and could not feasibly be based on an exhaustive review of all relevant fields.

More specifically CBS does suggest an awareness of behavioural function is particularly helpful, relevant and can likely increase precision with multiple challenges in psychedelic therapy, from helpful and unhelpful narcissism during the integration phase, to determining when and when not to intervene during the medication phase. Person-centred approaches can serve the function of allowing a participant to connect more deeply with the implicit subtleties of their inner experiencing, active-directive interventions can enable a participant to reconnect with the inner experiences that they unwittingly avoided, hopefully enabling more participants to transcend the barriers to reaching the integration phase. Thus, a containing awareness of behavioural function can also serve as a workable way to integrate coherently and context sensitively an array of psychedelic therapies (somatic or cognitive) that were previously separated on the level of form, but can be united on the level of function.

Ongoing psychedelic neuroscience findings may suggest an investigation into a broader spectrum of psychological flexibility. This could be useful to find more precise parameters for optimising functional psychological (and correspondingly behavioural) flexibility throughout the peaks and troughs of the three phases of psychedelic therapy.

Finally, high density, longitudinal idionomic data could elucidate how different contexts, interventions or processes can transfer helpful effects between the three phases of psychedelic-assisted therapy for improved and sustained outcomes.

## Data Availability Statement

The raw data supporting the conclusions of this article will be made available by the authors, without undue reservation.

## Ethics Statement

The studies involving human participants were reviewed and approved by Psychology Research Ethics Committee, School of Psychotherapy & Psychology, Regent's University London, Inner Circle, Regent's Park, London, NW1 4NS. The patients/participants provided their written informed consent to participate in this study. Written informed consent was obtained from the individual(s) for the publication of any potentially identifiable images or data included in this article.

## Author Contributions

HW wrote all drafts of the paper.

## Funding

This study was funded by the Regent's Centre for Relational Studies & Psychological Wellbeing.

## Conflict of Interest

The author declares that the research was conducted in the absence of any commercial or financial relationships that could be construed as a potential conflict of interest.

## Publisher's Note

All claims expressed in this article are solely those of the authors and do not necessarily represent those of their affiliated organizations, or those of the publisher, the editors and the reviewers. Any product that may be evaluated in this article, or claim that may be made by its manufacturer, is not guaranteed or endorsed by the publisher.
