# Erratum: A Spectrum of Selves Reinforced in Multilevel Coherence: A Contextual Behavioural Response to the Challenges of Psychedelic-Assisted Therapy Development

**DOI:** 10.3389/fpsyt.2021.832766

**Published:** 2021-12-24

**Authors:** Frontiers Production Office

**Affiliations:** Frontiers Media SA, Lausanne, Switzerland

**Keywords:** psilocybin-assisted psychotherapy, acceptance and commitment therapy, psychological flexibility, self-perspective taking, psychedelic, internal family systems, relapse prevention, integration

Due to a production error, the word “story” was misspelled in the article. A correction has been made to all instances of “storey” and “storeys” in the article. They have been corrected to “story” and “stories.”

Due to a production error, reference citation (1) was incorrectly linked in the **Introduction**, **line 3** and **line 5**. The citation on **line 3** has now been linked to the corresponding reference, while the citation on **line 5** has been un-linked.

The publisher apologizes for these mistakes. The original version of this article has been updated.

