# Supplementary material for: A Spectrum of Selves Reinforced in Multilevel Coherence: A Contextual Behavioural Response to the Challenges of Psychedelic-Assisted Therapy Development
Source: Front Psychiatry. 2021 Dec 7;12:727572. doi: 10.3389/fpsyt.2021.727572 (PMC8689588; doi:10.3389/fpsyt.2021.727572)
Supplement: Supplementary file 1 [file Data_Sheet_1.pdf]

Supplementary Materials to: *A Spectrum of Selves (SoS) Reinforced in Multilevel Coherence: A Contextual Behavioural response to the challenges of psychedelic-assisted therapy development*

Research Topic: *Can Psychedelic Therapies open a New Frontier in Mental Healthcare (Or Will the Bubble Burst?)*

See full article at https://www.frontiersin.org/articles/10.3389/fpsyt.2021.727572/full

Henry J. Whitfield © 2021

S1.0 CBS and varieties of Self

Since Hayes (1984), varieties of *self* in CBS have been broken down into three distinct categories. These three contrasting modes of *self* have since gained increasing empirical support (Moran et al., 2018), being increasingly informed by Relational Frame Theory (RFT), a lab-tested behavioural account of human language and cognition (See: Hayes, 1991; Moran & McHugh 2019; Hayes et al., 2021).

These three selves are:

1) A *self-as-story* or *Conceptualised self,* consists of evaluations and beliefs about who we are that we organise into a coherence, in order to create a consistent sense of self. A dark side of this behaviour is that we often choose behaviours congruent with our *self story* rather than choosing the life we want to live (McHugh et al., 2019, p.50). For example the thought "I'm no good at learning languages" initially acts as a momentary observation during a failure, and then operates with an aversive function that contributes to narrowing the person's behavioural repertoire, and reinforces behaviour that coheres with that self-story. Thus this reduces openness to new language learning opportunities as a person lives in line with the story they 'know' about themselves.

2) A *self-as-process* or *experiencing self,* by contrast is the momentary self-reference in the here and now of thoughts, emotions and other immediate experiences. A mode of '*selfing'* behaviour, consistent with mindful living, e.g. "Right now I notice I'm having the thought that I'm no good at languages". That is, a little more hypothetical distance between the thinker and the thought.

3) A *self-as-perspective* or *Transcendent self* is a self as a context, *containing* the content of one's observed experience. This points to the ability to step back from one perspective to another, fostering a sense of flexible perspective: e.g. a person experiences stepping back from the idea that their language ability is impaired, realising that this does not define them, but is a thought they can contain and of which they can act independently. This transcendent quality of stepping back from the perspective previously assumed to be *you*, to a broader context also aligns with many aspects of mystical experience (see Hayes, 1984. p.105).

S1.1 Self-as-hierarchy

Note that these three selves do not exist in isolation, but interrelate in a hierarchy. Note this is not a power-hierarchy in which one controls the next but an interdependence of interconnected selves

The *experiencing self* "feeds the conceptualized self" (McHugh et al 2019, p.111). That is, present moment experiences are often packaged into new and old stories. This relationship from self-as-process to content aligns with Damasio's (1999) tripartite theory of selves: the biological *protoself*'s stimuli are experienced by the *Core consciousness self* moment to moment (like self-as process) which in turn supplies the autobiographical *Extended Consciousness* with content to create a memory-based self (self-as-content).

Next, the Perspective-taking-self hierarchically frames and contains, the Experiencing and the Conceptualised selves. It is a self-behaviour that recognised both sense data and self-stories as momentary events, around which that self-perspective has choice. It provides a consistent context for all content (old and new), relating it all to a unity in the self (McHugh et al 2019, p.116).

Moving through these three selves a person's perspective is decreasingly influenced by the content it contains. Correspondingly, that content (thoughts, feelings, emotions framed with the self) influences behaviour less and less. The hypothetical "location" of psychological content moves from HERE and NOW (in self as content/process) to THERE and THEN (in self as perspective), thus the sense of self becomes more stable and secure (Barnes-Holmes et al., 2013, p.144).

An aim of ACT is to reverse this tendency of self-as-story to narrow a person's perspective and behavioural repertoires.

In alignment with the *containing* array of selves, many RFT lab studies have found superior empirical support (compared to other types of relational framing) for *hierarchical-framing* of the self (I contain this thought) to: 1) increase wellbeing (Moran & McHugh, 2019; Foody et al. 2013, vs. d*istinction-framing* (e.g. I am not this thought)), 2) increase distress tolerance (Gil-Luciano et al. 2017, vs. *deictic framing* (e.g. I-Here-Now)), 3) increase motivation (Murthy et al., 2019, vs. conditional framing (e.g. outcome focus)), and 4) increase cognitive performance (López-López & Luciano, 2017, compared to *deictic framing.*

Thus, hierarchical framing around the self has wide empirical support for increasing psychological flexibility.

S2.0 A convergence between the *selves of* CBS, Psychedelics and Neurobiology

In neurobiological terms, at least 'self-as-content' and 'self-as-process' are distinguishable in fMRI data (Farb et al. 2007) and highlight a convergence between, third-wave Contextual Behavioural Therapies, psychedelic qualitative research, and neurobiology (Hayes et al., 2020, p.35):

> …the data on psychedelics supports a model of psychological flexibility that emphasizes the central importance of the perspective-taking self or deictic 'I'. Consider, for example, how changes in the medial prefrontal cortex[1] increase a sense of transcendence and oneness, a reduction of self-narrative, and a greater capacity to experiencing ongoing emotions and sensations and their relation to the environment more deeply and meaningfully…

Hayes et al., (2020, p.36) even suggest "elevating the perspective-taking self to the one process that "rules them all.""

[1] 'a key part of our "narrative circuitry" that extends sense of self across time into a person with traits and aspirations']Hayes et al 2020

Other neurobiological data suggests that serotonergic psychedelics promote a similar neuroplasticity to that gained from mindfulness meditation (Heuschkel & Kuypers 2020), which may correspond with increased psychological flexibility as understood in CBS (Davis et al., 2020). In accordance, other data also show that transitions between varied brain-states requires less energy when LSD is ingested (Singleton et al., (in press); evidence for Relaxed Beliefs Under Psychedelics (REBUS)). Thus the psychological flexibility processes discussed and these pharmacological effects are likely to complement each other. Specifically targeting psychological flexibility processes in terms variations in self through hierarchical framing is helpful without psychedelics and apparently in alignment with neurobiological and experiential effects, occasioned by psychedelics.

S3.0 Further applications of a Spectrum of Selves

Having considered a new psychological flexibility model that places variation in self-perspective as the central and unifying process, let us briefly consider how it may be helpful for other challenges particular to psychedelic-assisted therapy.

S3.1 Malevolent Parts

Section 4.7 highlighted the possibility of participants encountering malevolent entities. Such an encounter can consist of an entity instructing a person to self-harm or even commit suicide. Whether the entity is considered a supernatural being or a subpersonality of the participant, parts work could again be a workable response. Watkins and Watkins (1988) successfully treated 'malevolent ego-states' in cases of Dissociative Identity Disorder (DID) using parts work largely consistent with IFS, finding that such malevolence can respond well to compassion:

> On the surface they [malevolent parts] are scornful and belligerant. Inwardly, like the angry child from whom they started, they yearn for acceptance and affection…The resulting change is not fusing but integration, a process in which the previous alters [parts] retain their unconscious sense of self. Their resistance to the procedure is greatly lowered because they are only being changed and do not feel they are being executed. (p.71).

Moreover, empirical data for the successful treatment of a trauma-focussed Schema Therapy (with parts work) for DID appears to be forthcoming (Huntjens et al., 2020). An assumption here is that treatments workable with extreme forms of dissociation may sometimes be helpful with the more extreme psychedelic therapy situations which appear to mirror aspects of psychosis. Hill (2021) highlights how daimonic encounters in the psychotic patients of Jung and Kalsched often serve the function of protecting a more innocent or conscious *part* from experiencing the inexperienceable (trauma).

S3.2 Healthy subclinical narcissism

This article has suggested it may be important to monitor the *function* of any new self-stories as they emerge. However, new self-referential content may be functional *or* dysfunctional. For instance, a positive self-story could help pull someone out of depressed inactivity, or trap them in a new comfort zone. Some data show that a grandiose subclinical narcissism predicted increased mental toughness which contributed to reduced depression symptoms (Papageorgiou et al., 2019). Positive self-referential stories need not be judged on the level of form but on their effects. During psychedelic integration it may be important to normalise new positive self-stories and not let them become new sources of shame indiscriminately. This example also points out how a radically

changing self-perspective could lead to new forms of shame that were not there pre-treatment. It may therefore be important for an integration therapist to normalise and pre-empt such complications arising from a fast changing sense of self, as well as monitoring and pre-empt when Positive self-referential stories have an unhealthy function (see Section 2.6 Inflated 'ego' and the narcissistic self).

S4.0 A Psychedelic Integration checklist*

1) What new behaviours since the psychedelic experience are spontaneously occurring? Note in which of the four domains they occur. (variation)
2) Brainstorm any new actions your new self-perspectives position you to take? (variation)
3) What values do these new behaviours point to? (selection)
4) Are you now more aware of values you wish to focus on or that have been neglected? (selection)
5) Which of these four life domains do these values touch? (notice reinforcing coherence/ punishing incoherence)
6) Are you more aware of behaviours that haven't served you? (de-selection) Which selves do these behaviours belong to? (awareness of self-perspective). In which domains have these self-perspectives/parts been active? (notice coherence/incoherence)
7) Which thoughts and emotions accompany or encourage the behaviours you wish to change?/which selves might you compassionately attend to? (awareness of inner contextual cues for unhelpful behaviour)
8) What experiential practices/therapeutic exercises could continue interaction with new implicit felt-senses that emerged during the psychedelic experience? Are there any autobiographical memories that remain aversive or emotionally charged? Is it worthwhile continuing to work with these somato-cognitively? (variation)
9) What practices/behaviours might help to nurture the new self-perspectives you have experienced and wish to develop or maintain? E.g. meditation, breathwork, journaling, writing a letter to your future stressed self (retention through exercises of self-perspective)
10) What behaviours on one domain-level might support behaviour change on another level? For example, do your relationships and community help with self-care? Can your self-care be in the service of being a better partner or contributing more to a community? Can you change your immediate environment to help encourage self-care practices? (coherence and context to serve retention)
11) Have any areas in the chart been neglected? If so, would it be useful to consider those neglected areas? E.g. lack of community, relational values, selfcare practices (additional choices to support retention).
12) Aim to make any behaviour change self-reinforcing by choosing behaviours that generate contexts/encourage the continuation of new behaviours. (retention)
13) Are there an difficult decisions worth considering that would change your daily environment/community/relationships/self-care towards the life you want to live? (retention via new context selection)
14) What would psychological/situational barriers make that decision difficult (bringing barriers into the work/variation in self perspective)

*Note that this checklist is an offering of possible options to be selected as appropriate/as they resonate in the therapeutic relationship, and need not be followed rigidly.
Particular therapeutic exercises can also be informed by this checklist using the skill sets that individual therapists have to hand. For example one method of 'brainstorming' for point number 2 could be to explore as a repetitive meditation a question like "**As your *compassionate self*** [insert self description that resonates with new felt sense of self], **tell me something you could really do"**
Such an exploration could also be focussed on an neglected area such as 'community', by specifically exploring potential behaviours for that domain as "something you could really do for community".

S5.0 A visual summary of the relationship between the different phases of therapy and their relationship to the outside world, as intended by this model.

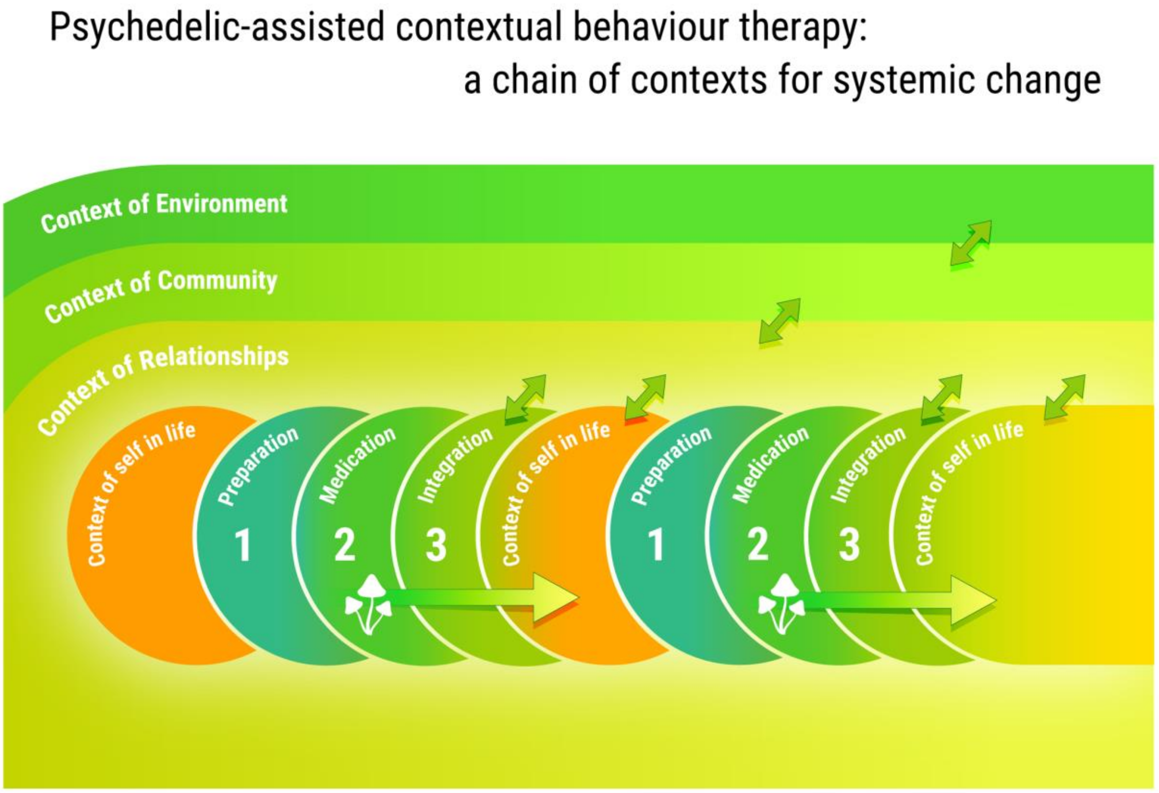

Henry J. Whitfield © 2021

Figure 4.

Note: In this view of psychedelic therapy, it is hoped that each context in the chain will influence the next, e.g. a greater awareness of self worth behaviours such as a contracted bodily posture or desire not to be seen, noticed during the preparation phase, may be carried into the medication phase where it could be examined with an increased capacity to feel and allow relevant self as content to surface and new perspectives to emerge. Any new self-perspectives can then be carried into the integration phase where it can be expressed behaviourally in life, and systemic changes can be intended to reinforce the new perspectives.

References:

Davis, A. K., Barrett, F. S., & Griffiths, R. R. (2020b). Psychological flexibility mediates the relations between acute psychedelic effects and subjective decreases in depression and anxiety. Journal of contextual behavioral science, 15, 39-45. https://doi.org/10.1016/j.jcbs.2019.11.004

Farb, N. A., Segal, Z. V., Mayberg, H., Bean, J., McKeon, D., Fatima, Z., & Anderson, A. K. (2007). Attending to the present: mindfulness meditation reveals distinct neural modes of self-reference. *Social cognitive and affective neuroscience*, *2*(4), 313-322. [https://doi.org/10.1093/scan/nsm030]

Foody, M., Barnes-Holmes, Y., Barnes-Holmes, D., & Luciano, C. (2013). An empirical investigation of hierarchical versus distinction relations in a self-based ACT exercise. *International Journal of Psychology and Psychological Therapy*, *13*(3), 373-388. [Book Link]

Gil-Luciano, B., Ruiz, F. J., Valdivia-Salas, S., & Suárez-Falcón, J. C. (2017). Promoting psychological flexibility on tolerance tasks: Framing behavior through deictic/hierarchical relations and specifying augmental functions. The Psychological Record, 67(1), 1-9. https://doi.org/10.1007/s40732-016-0200-5

Hayes, S. C. (1984). Making sense of spirituality. *Behaviorism*, 99-110. Vol 12, No. 2. https://www.jstor.org/stable/27759047 (last accessed 14/11/210

Hayes, S. C. (1991). A relational control theory of stimulus equivalence. *Dialogues on verbal behavior*, 19-40. [Book Link]

Hayes, S. C., Law, S., Malady, M., Zhu, Z., & Bai, X. (2020). The centrality of sense of self in psychological flexibility processes: What the neurobiological and psychological correlates of psychedelics suggest. *J Context Behav Sci*, *15*, 30-38

Hayes, S. C., Law, S., Assemi, K., Falletta-Cowden, N., Shamblin, M., Burleigh, K., ... & Smith, P. (2021). Relating is an Operant: A Fly Over of 35 Years of RFT Research. Perspectivas em Análise do Comportamento. https://doi.org/10.18761/PAC.2021.v12.RFT.02

Hill, S., (2021), Chapter 5 in Read., T & Papaspyrou, M., *Psychedelics & Psychotherapy: The Healing Potential of Expanded States.* Inner Traditions Bear and Company

Huntjens, R. J. C., Rijkeboer, M. M., & Arntz, A. (2020). Schematherapy in DID: treatment length and related studies on dissociative amnesia. European journal of psychotraumatology, 11(1), 1711638. https://doi.org/10.1080/20008198.2020.1711638

López-López, J. C., & Luciano, C. (2017). An experimental analysis of defusion interactions based on deictic and hierarchical framings and their impact on cognitive performance. The Psychological Record, 67(4), 485-497. https://doi.org/10.1007/s40732-017-0250-3

McHugh, L., Stewart, I., & Almada, P. (2019). A contextual behavioral guide to the self: Theory and practice. New Harbinger Publications.

Moran, O., Almada, P., & McHugh, L. (2018). An investigation into the relationship between the three selves (Self-as-Content, Self-as-Process and Self-as-Context) and mental health in adolescents. *Journal of Contextual Behavioral Science*, *7*, 55-62. [https://doi.org/10.1016/j.jcbs.2018.01.002]

Moran, O., & McHugh, L. (2019). Patterns of relational responding and a healthy self in older adolescents. Journal of Contextual Behavioral Science, 12, 74-80. https://doi.org/10.1016/j.jcbs.2019.02.002

Murthy, V. E., Villatte, M., & McHugh, L. (2019). Investigating the effect of conditional vs hierarchical framing on motivation. Learning and Motivation, 65, 33-42. https://doi.org/10.1016/j.lmot.2018.11.002

Papageorgiou, K. A., Denovan, A., & Dagnall, N. (2019). The positive effect of narcissism on depressive symptoms through mental toughness: Narcissism may be a dark trait but it does help with seeing the world less grey. *European Psychiatry*, *55*, 74-79. https://doi.org/10.1016/j.eurpsy.2018.10.002

Singleton S. P.,. Luppi, A. I., Carhart-Harris, Cruzat, J.D., Roseman L.E.F., Deco, G.D., Morten L., Kringelbach Stamatakis E.A., & Kuceyeski, A., (in press) LSD flattens the brain's energy landscape: evidence from receptor-informed network control theory. bioRxiv. https://doi.org/10.1101/2021.05.14.444193

Watkins, J. G., & Watkins, H. H. (1988). The management of malevolent ego states in multiple personality disorder. Dissociation, 1(1), 67-71.
